# Citizen-science detects the arrival and establishment of *Branchiomma luctuosum* (Grube, 1870) (Annelida: Polychaeta: Sabellidae) in Albania

**DOI:** 10.3897/BDJ.8.e54790

**Published:** 2020-08-05

**Authors:** Valentina Tanduo, Aleksander Golemaj, Fabio Crocetta

**Affiliations:** 1 Department of Integrative Marine Ecology, Stazione Zoologica Anton Dohrn, Villa Comunale, I-80121, Napoli, Italy Department of Integrative Marine Ecology, Stazione Zoologica Anton Dohrn, Villa Comunale, I-80121 Napoli Italy; 2 L. Deshmoret, Rr. Petrit Bisha, Ap 544 Vlore, Albania L. Deshmoret, Rr. Petrit Bisha Ap 544 Vlore Albania

**Keywords:** Mediterranean Sea, social media data mining, bioinvasions, shipping, Sabellidae

## Abstract

The invasive fan worm *Branchiomma luctuosum* (Grube, 1870), originally described from the Red Sea, is first reported here from the Albanian coasts, based on records held in Vlora Bay, a locality near to Valona harbour and Narta Lagoon. Possible pathways of arrival in the area are uncertain. However, species’ larval ecology and life-history traits suggest a secondary spreading through shipping. Social media data mining allowed the confirmation of its establishment in the area, with specimens showing high densities in shallow waters on artificial hard substrates and to backdate its arrival in the area since at least November 2016. Citizen science continues supporting marine biology in the Mediterranean area, especially in countries where proper field studies and research projects are still limited.

## Introduction

The Mediterranean Sea is considered a hotspot of biodiversity, with around 17,000 accepted species, of which around one fourth is endemic (Coll et al. 2010, Bianchi et al. 2012). This diversified biota is threatened by a number of anthropogenic factors, including the introduction of non-indigenous species (NIS), that may compete with local fauna and affect biodiversity maintenance (Wallentinus and Nyberg 2007, Katsanevakis et al. 2014). NIS invasion in the Mediterranean Sea is mainly due to Lessepsian migration through the Suez Canal, connecting two biogeographic provinces (the north-eastern Atlantic-Mediterranean and the Indo-Pacific), and commercial shipping traffic, with species transported worldwide as fouling or through ballast waters. A high number of species are also introduced either deliberately or not through aquaculture (Katsanevakis et al. 2014, Galil et al. 2017, Zenetos et al. 2017).

Despite Descriptor 2 of the EU Marine Strategy Framework Directive (European Commission 2008) suggesting to maintain the number of NIS at levels that do not adversely alter the ecosystems, NIS invasion in the Mediterranean Sea is an increasing phenomenon, with the most recent review enumerating 821 taxa, of which 613 have been established (Zenetos et al. 2017). This called the attention of the local scientific community, that tried to monitor this through a wide list of databases, inventories and horizon scannings (e.g. Tsiamis et al. 2015, Tsiamis et al. 2020, Crocetta et al. 2017, Zenetos et al. 2017). In recent years, the involvement of citizen scientists also further contributed to early detection and mapping of NIS species (e.g. Delaney et al. 2008, Crall et al. 2010, Zenetos et al. 2013, Langeneck et al. 2019, Paz-Sedano et al. 2019).

Amongst NIS species invading the Mediterranean Sea, the phylum Annelida ranks high (Zenetos et al. 2017). Within Annelida, the family Sabellidae Latreille, 1825 includes sedentary tube-building polychaetes commonly found in fouling communities (Keppel et al. 2015, Khedhri et al. 2017) and, within Sabellidae, the genus *Branchiomma* Kölliker, 1858 includes about 30 species, characterised by paired compound radiolar eyes and stylodes (epithelial flaps) on the outer surface of the radiolar axes of the crown (Tovar-Hernández and Knight-Jones 2006, Capa et al. 2013, Çinar 2013, WoRMS 2020). *Branchiomma* is a widespread genus whose taxa live in sheltered shallow waters worldwide and is represented in the Mediterranean Sea by nine species, of which three are aliens, namely *Branchiomma bairdi* (Mclntosh, 1885), originally described from Bermuda, *Branchiomma boholense* (Grube, 1878), native to the Indo-Pacific, and *Branchiomma luctuosum* (Grube, 1870), originally described from the Red Sea (Licciano and Giangrande 2008, Keppel et al. 2015, Khedhri et al. 2017, Del Pasqua et al. 2018). Despite records of the complex constituted by the two former species dating back to at least 1927, misidentifications amongst them and a general unresolved taxonomy make difficult the delimitation of their distribution in the Mediterranean Sea (Del Pasqua et al. 2018, Langeneck et al. 2020). On the other hand, since the first sighting of *B. luctuosum* from Lago Lucrino (Naples, Tyrrhenian Sea) in the late 1970s (Giangrande 1989, Knight-Jones et al. 1991: statements/records based on the material collected by C.N. Bianchi and partially published in Bianchi 1983), records of this latter species proliferated in the Mediterranean Sea due to its characteristic colour pattern and straightforward identification, being subsequently observed in several localities in Italy (e.g. Giangrande 1989, Sordino and Gambi 1992, Gambi et al. 1998, Giangrande and Gambi 1998, Licciano et al. 2002, Mastrototaro et al. 2004, Cosentino et al. 2009, Tempesti et al. 2020, Licciano et al. 2012, Bianchi et al. 2018, Langeneck et al. 2020, Langeneck et al. 2020) and, beyond Italian waters, in Greece (Arvanitidis 2000, Zenetos et al. 2008, Zenetos et al. 2018), Turkey (Çinar et al. 2006), Cyprus (Çinar 2005) and Spain (El Haddad et al. 2008, El Haddad et al. 2012). Indeed, such a high spread in Italian waters and absence of records from several nearby countries may suggest that its distribution could be at least partially overlooked due to absence of field studies. We here confirm this statement by first reporting the presence of the invasive fan worm *B. luctuosum* in Albania (Adriatic Sea).

## Materials and methods

The present record falls within the framework of an ongoing project that aims at monitoring the marine biodiversity in Albania. In particular, a folder of photos and videos of an unknown annelid species living in the shallow waters of Vlora Bay, Albania (40°45’72’’N, 19°39’80’’E) were posted by one of the authors (A.G.) in October 2019 on the Facebook group Regjistri Elektronik i Specieve Shqiptare (Electronic register of Albanian species: https://www.facebook.com/groups/220793668293252). Soon after its identification, additional photos uploaded on Facebook by the author of the present finding and dealing with the marine biota of the Vlora Bay were screened in the two Facebook profiles maintained by him (Albanian Mollusca: https://www.facebook.com/connus74/; Aleksander Golemaj: https://www.facebook.com/conus74) to search for a possible presence of the species in the area prior to the post noted in the origin. Finally, a careful bibliographic research was done to evaluate the distribution of this species in the Mediterranean Sea.

## Results

**Figure 1.**
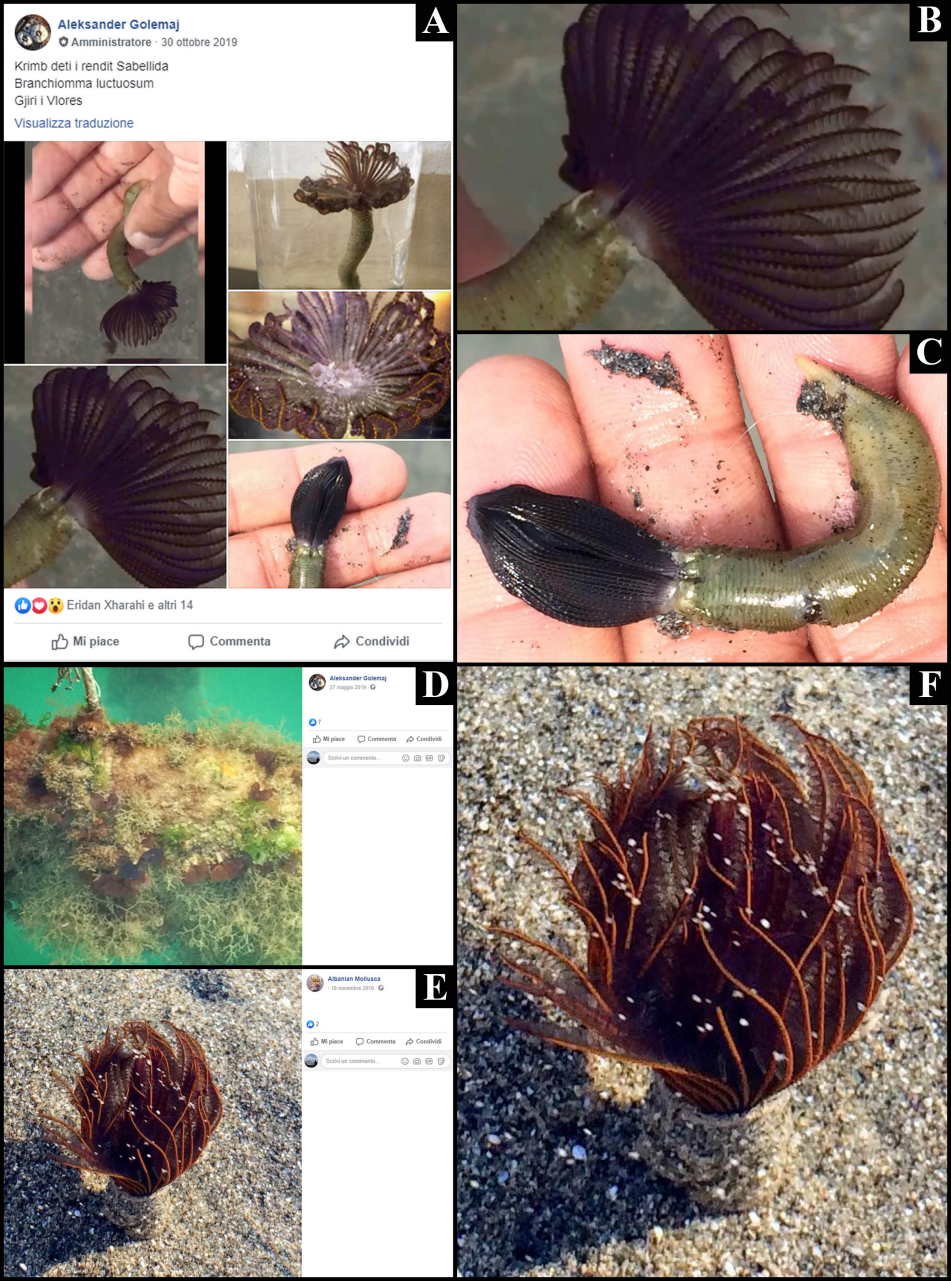
The sabellid polychaete *Branchiomma luctuosum* (Grube, 1870) from Vlora Bay (Albania, Adriatic Sea). A. Folder of photos and videos posted in October 2019 on the Facebook group Regjistri Elektronik i Specieve Shqiptare; B-C. A magnification of selected photos; D. An artificial hard substrate dominated by *B. luctuosum* (red circles) and the spaghetti bryozoan *Amathia verticillata* (delle Chiaje, 1822), posted in May 2019 on the Facebook® profile of Aleksander Golemaj; E-F. The photo backdating the presence of *B. luctuosum* in Albania, posted in November 2016 on the Facebook® profile of Albanian Mollusca, and its magnification.

**Figure 2.**
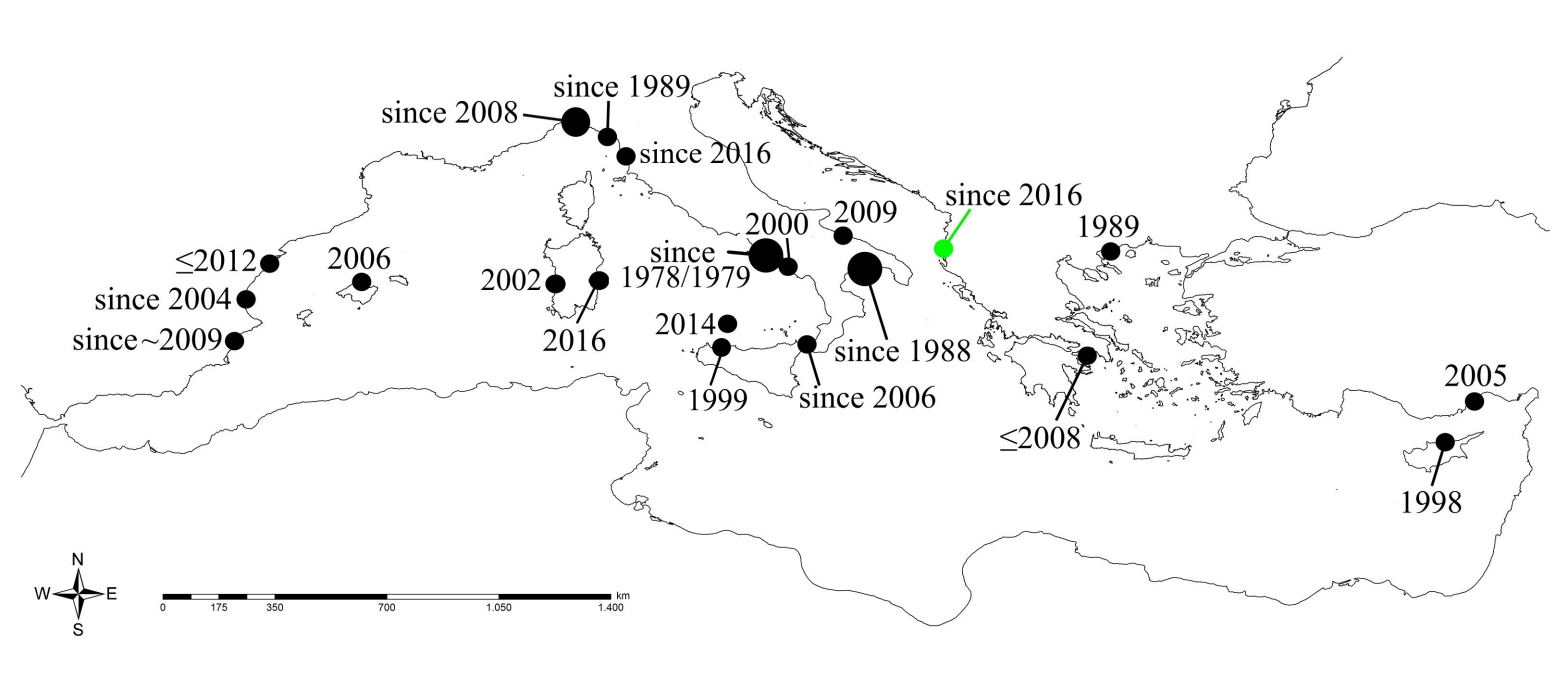
Map of the known records of *Branchiomma luctuosum* (Grube, 1870) in the Mediterranean Sea, with first year of collection per area. Larger dots correspond to the presence of more than one locality known per wider geographic area. Green dot highlights the present sighting. Localities, coordinates and references reported in Table S1 (arranged for first finding date per paper).

The unknown annelid specimens were identified as *Branchiomma luctuosum* (Grube, 1870) based on their large sizes, external appearance/general colour pattern with a brownish/greenish body and a dark velvet crown and absence of macrostylodes. These characters altogether make this species easier to identify with respect to the other alien and native *Branchiomma* species living in the Mediterranean Sea, even from underwater photographs and videos. This is confirmed by the fact that the species has already been widely recorded in the Mediterranean Sea based on ROV observations and visual census (Matarrese et al. 2006, El Haddad et al. 2012, Katsanevakis et al. 2020). Facebook® data mining allowed us to confirm its establishment in the area, with specimens showing high densities in shallow waters (up to 1 m depth) on artificial hard substrates of local marinas, and to backdate the arrival of this species in Albania since at least November 2016 (Fig. 1). Finally, screening of the published literature revealed that this species was never recorded from Albania and that its presence in the entire Adriatic Sea was only known so far on the basis of a single sighting from Brindisi Harbour (Italy) (Mikac 2015, Suppl. material 1, Fig. 2).

## Discussion

The present paper confirms the presence of *Branchiomma luctuosum* in the Adriatic Sea and supports the hypothesis that, after 50 years from its arrival, this taxon is well established in the Mediterranean region, as already suggested in recent literature reviews (e.g. Simboura and Nicolaidou 2001, Çinar et al. 2006, Zenetos et al. 2017, Servello et al. 2019) and, at the same time, it highlights the possibility that *B. luctuosum* could even further spread in the Mediterranean basin. In addition, the area where the species has been found in this study is a shallow water bay nearby Valona harbour and Narta Lagoon. It is well known that ports, estuaries and other brackish environments host a relatively low and restricted biodiversity and are generally characterised by multiple biotic and abiotic stressors that may alter the local biota and favour the presence of empty niches that could be colonised by alien species (Occhipinti-Ambrogi and Savini 2003, El Haddad et al. 2012). In this view, Vlora Bay can be easily considered a hot-spot for alien species introduction in Albania and the entire Adriatic Sea, as also confirmed by previous records of NIS species in the area (Gerovasileiou et al. 2017).

No certainties occur regarding a possible pathway of arrival of *B. luctuosum* in the Vlora Bay. This taxon is a hermaphrodite with a short life cycle, a rapid growth and a high fecundity (Licciano et al. 2002, Mastrototaro et al. 2014), circumstances that can indeed facilitate its expansion to areas with favourable conditions for settlement. In addition, its larvae are lecithotrophic, a feature that would allow its spreading through maritime vessels and ballast waters (Licciano et al. 2002, El Haddad et al. 2008). However, its spread in Vlora Bay could also have been achieved through recreational boating, a vector that has a pivotal role in facilitating and accelerating secondary spread of species with lecithotrophic larvae (Ulman et al. 2017). In agreement with that, despite of its Indo-Pacific origin that led some authors in the past to consider *B. luctuosum* as a Lessepsian element (Mastrototaro et al. 2014, Matarrese et al. 2006), the shipping pathway has already been suggested as the most probable vector even for its arrival in the Mediterranean Sea (El Haddad et al. 2008), supported by the fact that early records were registered from the central Mediterranean Sea and that the species is still undetected in the easternmost parts of the basin.

Generally, when an alien species colonises a new area, it could enter in competition with the local biota; in the case of *B. luctuosum*, its major antagonist may be the Mediterranean annelid *Sabella spallanzanii* (Gmelin, 1791), that occupies the same niche (Mastrototaro et al. 2014). However, there is still no particular evidence of competition nowadays; nevertheless, some authors (e.g. Licciano et al. 2002, El Haddad et al. 2008) observed that *B. luctuosum* settled on the base of the tubes of *S. spallanzanii* or amongst specimens of *Mytilus galloprovincialis* (Lamarck, 1819), *Actinia equina* (Linnaeus, 1758) and *Balanus perforatus* Bruguière, 1789, or as an epibiont of the ascidian *Styela plicata* (Lesueur, 1823) and the crustacean *Maja squinado* (Herbst, 1788) (Licciano et al. 2002, El Haddad et al. 2008).

Finally, worth a mention, the number of alien species detected in recent years in the Mediterranean Sea is also increasing thanks to citizen science projects and related field surveys (e.g. Giovos et al. 2019, Kleitou et al. 2019, Paz-Sedano et al. 2019, amongst others), and this also holds true for Albania (see discussions in Gerovasileiou et al. 2017). However, there is also a constant need to monitor distribution, establishment and propagation dynamics over time in order to understand if particular alien species are involved in interactions with other local organisms or can establish breeding populations. The active engagement of citizens and scuba divers in projects and scientific productions may constitute a practical management strategy of NIS and may offer accurate information about target species.

## Supplementary Material

31017BA7-FAF5-58EE-97BC-5AE0E94A2EB0 10.3897/BDJ.8.e54790.suppl1 Supplementary material 1 Supplementary-Citizen-science detects the arrival and establishment of B. luctuosum in Albania Data type Record of Branchiomma luctuosum in the Mediterranean Sea File: oo_413312.xlsx https://binary.pensoft.net/file/413312 Valentina Tanduo, Aleksander Golemaj, Fabio Crocetta
